# Fibre Optic System for Monitoring Rotational Seismic Phenomena

**DOI:** 10.3390/s140305459

**Published:** 2014-03-19

**Authors:** Anna Kurzych, Leszek R. Jaroszewicz, Zbigniew Krajewski, Krzysztof P. Teisseyre, Jerzy K. Kowalski

**Affiliations:** 1 Institute of Applied Physics, Military University of Technology, 2 Gen. Sylwestra Kaliskiego St., 00-908 Warsaw, Poland; E-Mails: akurzych@wat.edu.pl (A.K.); zkrajewski@wat.edu.pl (Z.K.); 2 Institute of Geophysics, Polish Academy of Sciences, 64 Księcia Janusza St., 01-452 Warsaw, Poland; E-Mail: kt@igf.edu.pl; 3 m-Soft Ltd., 9-4 Sotta St., 02-790 Warsaw, Poland; E-Mail: jkk@m-soft.pl

**Keywords:** fibre optic Sagnac interferometer, rotational seismic phenomena, continuously monitoring, remote control

## Abstract

We outline the development and the application in a field test of the Autonomous Fibre-Optic Rotational Seismograph (AFORS), which utilizes the Sagnac effect for a direct measurement of the seismic-origin rotations of the ground. The main advantage of AFORS is its complete insensitivity to linear motions, as well as a direct measurement of rotational components emitted during seismic events. The presented system contains a special autonomous signal processing unit which optimizes its operation for the measurement of rotation motions, whereas the applied telemetric system based on the Internet allows for an AFORS remote control. The laboratory investigation of such two devices indicated that they keep an accuracy of no less than 5.1 × 10^−9^ to 5.5 × 10^−8^ rad/s in the detection frequency band from 0.83∼106.15 Hz and protect linear changes of sensitivity in the above bandpass. Some experimental results of an AFORS-1 application for a continuous monitoring of the rotational events in the Książ (Poland) seismological observatory are also presented.

## 1. Introduction

Rotational seismic phenomena have been investigated theoretically for over thirty years [1]. The interest in them has been initiated by observation of phenomena which appeared after the earthquakes, where strange, rotational deformations of monuments or tombs gave reason to study the mechanism of their formation [2]. Since the classical approach to seismic waves presented in seismological textbooks excludes the possibility of rotational movements'existence, the observed phenomena were interpreted by an interaction of seismic waves with a compound structure of objects they pass through. Independently from the above mentioned conventional view, the existence of seismic rotational phenomena in grained rocks in a form of rotational events, as well as seismic rotational waves [3] has been studied in a few centres all over the world. In further consideration this property has been extended to rocks with microstructures or defects [4,5] or even any internal structure [6–8].

When rotation, present in the seismic field, is measured with the use of a special array or set of conventional seismometers, results are prone to disturbances caused by the high sensitivity of such instruments to linear (translational) motions [9,10]. In consequence new instruments for measuring the rotational components of ground motion are indispensable. In our opinion, the devices based on the Sagnac effect [11] seem to be the most promising. There are known other constructions which detect changes of rotation, but only those based on Sagnac effect principle lack inertia and, therefore they detect the rotation itself, this being a main advantage of such equipment. We distinguish two types of required rotation measurement systems: a ring laser [12], and a fibre-optic seismometer [13–15], both based on a technical implementation of the Sagnac interferometer.

In this paper we summarize our experiments on the construction, investigation and field application of the fibre optic interferometric device named Autonomous Fibre-Optic Rotational Seismograph (AFORS) [16]. In our opinion it is one of the limited examples of practical implementation of a fibre optic system in an interferometric configuration which has properly and continuously worked in the field for more than three years. A high accuracy, compactness, as well as a special signal processing unit ensuring its autonomous operation are AFORS'main advantages. Moreover, AFORS can be monitored, as well as remotely controlled via the Internet, thus we are convinced of the great usefulness of such a system for the investigation of seismic rotational phenomena. At the beginning of this paper, we describe the construction and the main parameters of AFORS with an indication of its most important advantages. In the next part we show examples of measurements recorded by AFORS-1 installed at the seismological observatory in Książ, Poland, and, finally, in the conclusion we present the main challenges for our system.

## 2. Construction and Investigation of the Autonomous Fibre-Optic Rotational Seismograph

We used a fibre optic Sagnac interferometer in a so-called minimal configuration developed for a fibre-optic gyroscope (FOG) [17] operating in open-loop architecture with digital data processing [18] as a basis for the construction of our rotational seismometer. The main reason for such an approach is the fact that the rotational phenomena (Ω are recorded as sudden changes of a rotation rate with an amplitude directly calculated from detected Sagnac phase shift (Δφ as [19]:

(1) $$\Omega =S_{o}\cdot \Delta \varphi =\frac{\lambda c}{4\pi RL}\cdot \Delta \varphi$$

where S_0_ is the optical constant of interferometer depending on the used wavelength— λ velocity of light in the vacuum—c, the length of fibre in the sensor loop—L and the sensor coil radius—R.

**Figure 1.**
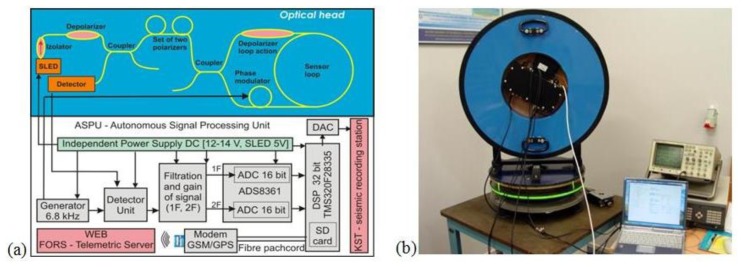
(**a**) General schema of the AFORS: upper—the optical head (generation of the Sagnac phase shift proportional to the measured rotation rate Ω), bottom—Autonomous Signal Processing Unit (rotation calculation and recording); (**b**) General view of AFORS-1 during calibration and scaling procedure at Military University of Technology, Warsaw, Poland.

The technical optimization of a constructed sensor gives the optical head of AFORS according to the schema presented in the upper part of Figure 1a. Application of a wideband, low coherence superluminescent diode SLED (Exalos, Schlieren, Switzerland; with a bandwidth of 31.2 nm, a central wavelength of 1,326.9 nm and an optical power of 20.8 mW) gives a possibility to minimize a polarization influence on the system operation (polarization fading in sensor loop) by achieving light depolarization in a sensor loop as was previously wide described [20,21]. Moreover, we applied the depolarizer (Phoenix Photonics with DOP <5% and insertion loss 0.20 dB) to obtain entirely depolarized light before it passes through the set of polarizers. The system uses two X-type couplers (Phoenix Photonics, Birchington, UK; with 0.2 dB insertion loss), one as an input/output way from a sensor loop and an additional one to separate a returned beam on a detector, and a fibre optic isolator (FCA, Niepołomice, Poland; with 0.34 dB insertion loss and 39 dB isolation) for SLED protection. Next, the set of two fibre-optic polarizers mounted in-line (Phoenix Photonics with extinction ratio 43 dB and 0.45 insertion loss each) with the total extinction ratio higher than 80 dB enables a true single mode operation of the whole system and guarantees that only nonreciprocal effect in system is the Sagnac effect. Moreover, a 0.63 m diameter sensor loop has been made from a special composite material with permalloy particles for shielding the sensor from any external magnetic field. A long length of SMF-28e + (Corning, Steuben, NY, US) fibre wound in a double-quadrupole mode [22] with a 0.2 mm Teflon insulation between each fibre layers is used for the thermal stabilization of the sensor's work, or expected 2–4 degree per day temperature fluctuation in seismic observatories. System optimization performed for AFORS (15 km fibre length with attenuation equal to 0.436 dB/km or 0.451 dB/km in sensor loops) allows for a theoretical sensitivity equal to 1.97 × 10^−9^ rad/s/Hz^1/2^ and 2.46 × 10^−9^ rad/s/Hz^1/2^ in quantum noise limitation, respectively for AFORS-1 and AFORS-2. The above mentioned difference between two constructed devices is connected to their total optical loss which is equal to 13.33 dB and 14.47 dB, for AFORS-1 and AFORS-2, respectively.

In contrast to FOG, a rotational seismometer needs information only about a rotation rate which is obtained directly from the measurement of the Sagnac phase shift, hence, a special autonomous signal processing unit ASPU (ELPROMA Ltd., Warsaw, Poland) has been applied. The ASPU (lower part of Figure 1a) enables the detection rotation rate (Ω) by properly selecting and processing the first (A_1ω_) and second (A_2ω_) amplitude of harmonic output signal (u(t)), according to the relation [16]:

(2) $$\Omega =S_{o}\cdot \arctan[S_{e}\cdot u(t)]=S_{o}\cdot \arctan[S_{e}\cdot \frac{A_{1\omega }}{A_{2\omega }}]$$

where S_e_—the electrical constants related with parameters of applied components and obtained during the sensor calibration together with determining the S_o_ constant. Moreover, the Equation (2) applied the arcus tangent function extended to the four quarters (−π, π which don't include discontinuity) after Fourier transform. As one can see, the ASPU realizes synchronous detection in a digital form, where 32 bit DSP (TMS320F28335, Texas Instruments, Dallas, TX, US) has implemented all the needed processing procedures according to the calculated rotation rate in time, getting it properly stored in a SD card, as well as sending it via fibre patch-cord (750 m long) to a GSM/GSP modem connected with WEB special FORS-Telemetric Server. The basic detection time, obtained from applied quartz oscillator in ASPU is equal to 4.7104 ms which corresponds to a 106.15 Hz detection frequency band. In order to adjust a narrower detection band, the multiplication of the basic detection time is possible. From a digital point of view the maximum multiplication equals 2^7^ and was used, which corresponds with 0.83 Hz detection frequency band. Such approach enables to detect rotational events in expected frequency range which is suggested as about 0.5–100 Hz by seismological investigation [23]. Moreover, additional DAC connected with a DSP unit is used as an interface for sending the obtained data to a standard seismic recording station, called KST, in an analogue form. Since the whole system allows for monitoring rotation events with their recording, the name Fibre-Optic Rotational Seismograph is properly used in our opinion.

**Figure 2.**
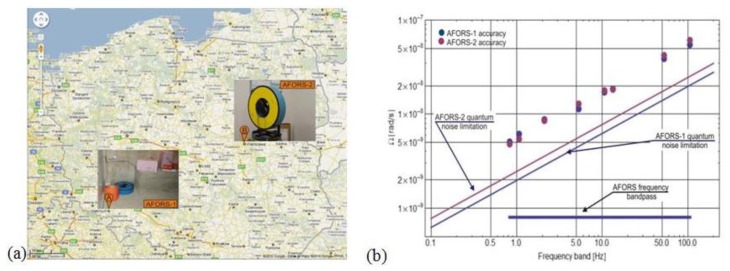
(**a**) GOOGLE map with current device locations; (**b**) The accuracy measured in Warsaw, Poland for the chosen detection frequency band for AFORS-1 and AFORS-2.

The calibration procedure of AFORS is based on the measurement of the Earth's rotation in Warsaw, Poland, *i.e.*, Ω_E_ = 9.18 deg/h = 4.45 × 10^−5^ rad/s for ϕ = 52°20′ [16]. During this process the AFORS was mounted vertically on a special rotational table (Figure 1b) and then the sensor loop was turned precisely to the North, East, West and South direction. The maximal values plus/minus of the measured rotation signal are observed when the senor is directed N-S because the sensor loop plane is perpendicular to the vector component of the Earth's rotation Ω_E_. For the E-W direction the measured rotation value should equal to zero because in this position the sensor plane is collinear with the Earth's rotational axis. Since it is hard to precisely position the system at the W-E directions based on looking for zero signals, those directions were obtained as the middle of the positions between the positions for maximal signals (N-S directions) with a limited angular accuracy of about 0.5 deg. Additionally, long-term signal averaging for about 10 night hours has been used to minimize drift phenomena. The set of the above four signals is used next for the calculation value of S_0_ and S_e_ parameters for a given AFORS. For two existing systems, the following values of the above constants were obtained: S_0_ = 0.043 s^−1^, S_e_ = 0.0144 for AFORS-1 and S_0_ = 0.059 × s^−1^, S_e_ = 0.0134 for AFORS-2. In the next step the accuracy of a constructed AFORS device has been checked by the measurement of the noise levels for given devices [16]. However, this work performed at a university located in Warsaw, Poland (Figure 2a could give only limited information about the system accuracy because of town noises. The obtained accuracy is at a level of 5.07 × 10^−9^/4.81 × 10^−9^ rad/s–5.51 × 10^−8^/6.11 × 10^-8^ rad/s (for AFORS-1/AFORS-2), respectively, for the lower and higher working detection frequency band, as shown in Figure 2b. It should be noted that the linear dependence of AFORS accuracy on the detection frequency band is an advantage of this system, taking into account the expected frequency characteristics of the rotational component of seismic waves [23].

Finally, the word ‘autonomous’ used in AFORS acronym should be explained. Our devices use the FORS-Telemetric Server (please use http://fors.m2s.pl with login and password: AFORSbook, for open access to the system) for data storage and for monitoring the work of the AFORS. Since ASPU contains a GSM/GPS module, its work can be remotely controlled via a server. ASPU also contains an independent power supply for all electronic components of the system, hence the AFORS became fully autonomous and mobile systems. The applied technology allows the possibility for remote (via Internet) control and changes of all the electronic parameters of the ASPU for a given AFORS. This remote control may be compared to the software upgrade, as well.

**Figure 3.**
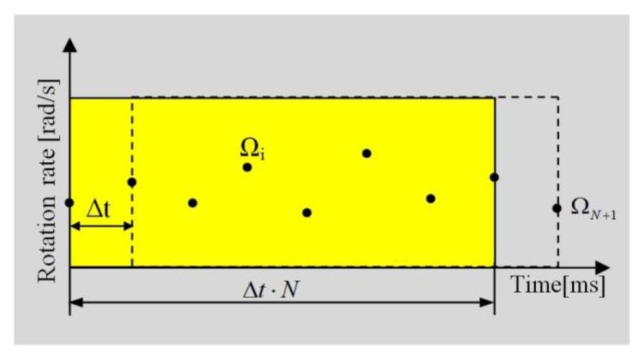
The general schema for choosing the moment of data recording used by AFORS. After a discrete time Δt = 4.7104 ms, the next sequence for data calculation is chosen.

For the first field test, at the beginning of July 2010, the AFORS-1 unit was installed in the seismological observatory in Książ, South-Western Poland (Figure 2a) and has been working continuously up to now. For more than three years during the system investigation, the main problem was connected with the proper method of occurrence selection for data recording. Generally, the system should start to record the data in the moment when a detected rotation rate has some assumed value above a continuously recorded level. Since a continuously recorded signal has slow changes with drift phenomena, choosing the absolute level of a rotation rate as the beginning point for event recording is the wrong method, so in order to select the starting point for automatic recording of rotational events, the following methodology has been applied:

The recursive procedure is used for gaining the level of detection. Firstly, for an assumed number of samples N (from our experience N equals 64), the mean and deviation of the mean (for a recorded rotation rate Ω_i_ represented as dots in Figure 3) are calculated:

(3a) $$\Omega_{av}=\frac{\overset{N}{\underset{i}{\text{∑}}}\Omega_{i}}{N}$$

(3b) $$\Delta \Omega_{av}=\frac{\overset{N}{\underset{i=1}{\text{∑}}}\left|\Omega_{i}-\Omega_{av}\right|}{N}$$

For the next recording points (Ω_N+1_ in Figure 3) the consecutive formulas to count the above mentioned volumes are used:

(4a) $${\Omega^{\prime }}_{av}=\Omega_{av}\cdot \frac{N-1}{N}+\Omega_{N+1}\cdot \frac{1}{N}$$

(4b) $$\Delta {\Omega^{\prime }}_{av}=\Delta \Omega_{av}\cdot \frac{N-1}{N}+\left|\Omega_{av}-\Omega_{N+1}\right|\cdot \frac{1}{N}$$

Finally, the detection condition is given by the formula:

(5) $$|\Omega_{av}^{'}-\Omega_{N+1}|>\Delta \Omega_{av}^{'}\cdot k_{L}$$

where *k_L_* is the chosen detection threshold. When the above Equation (5) is met we start recording the rotational seismic events with the AFORS.

## 3. Examples of Rotational Events Recorded by AFORS-1 in the Seismological Observatory in Książ, Poland

**Figure 4.**
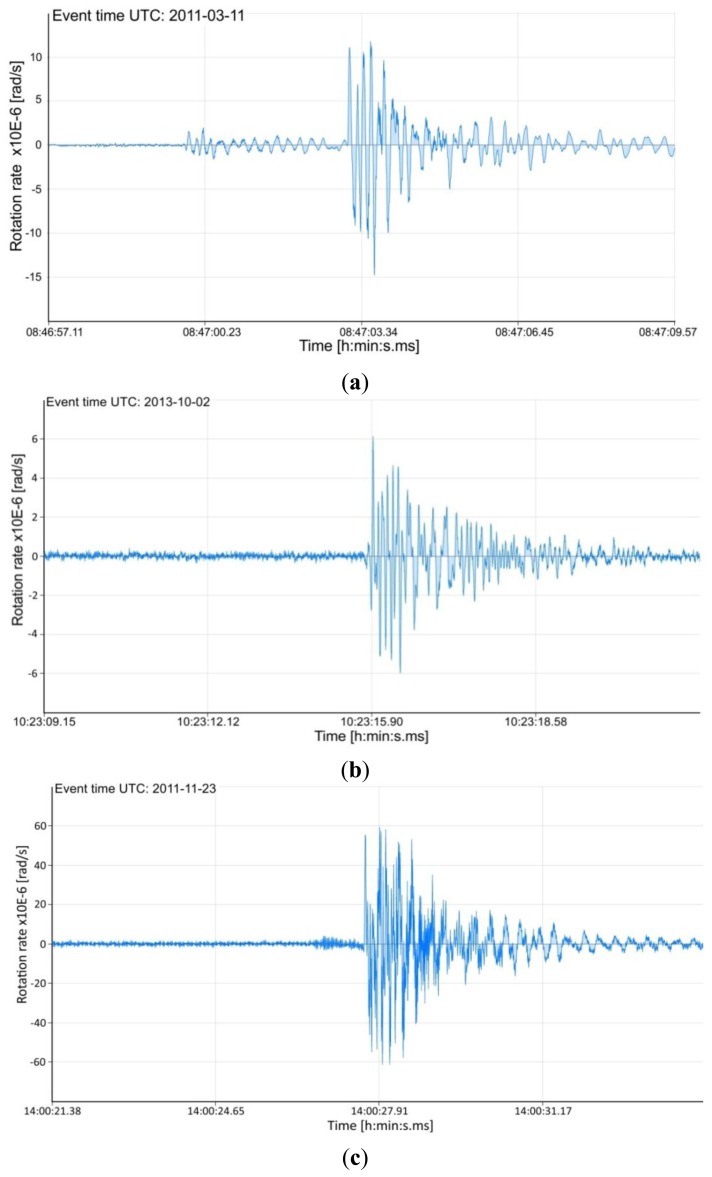
Examples of the rotational seismic events recorded in Książ, Poland, in 2011–2013 by AFORS-1with different detection frequency bands ΔB; (**a**) Plot of event recorded on 11 March 2011 at 8 h 46 min. (after the Honshu earthquake M = 9.0 on 11 March 2011 at 5 h 46 min. 23 s UT); (**b**)–(**c**) Selected events recorded in the last two mounts which source is the regional seismic mining events of the magnitude of *M* = 2.3–3.3, which occurred in the Lubin area (the Legnica-Głogów Copper Mining District, in the Western Poland).

The plots shown in Figure 4 present the examples of the rotation seismic events recorded by the AFORS-1 system installed at the seismological observatory in Książ, Poland. The AFORS-1, located in the huge undergrounds of the Książ castle (see Figure 2a) is connected with the FORS-Telemetric Server via a GPS/GSM external antenna, as well as with the KST recording system located in the same place. The set of two rotational seismometers positioned perpendicularly (each comprises Two Anitparallel Pendulum Seismometers-TAPS) constructed by the Institute of Geophysics [24], are aslo mounted and connected with the KST recording system (see left photo in Figure 2a).

**Figure 5.**
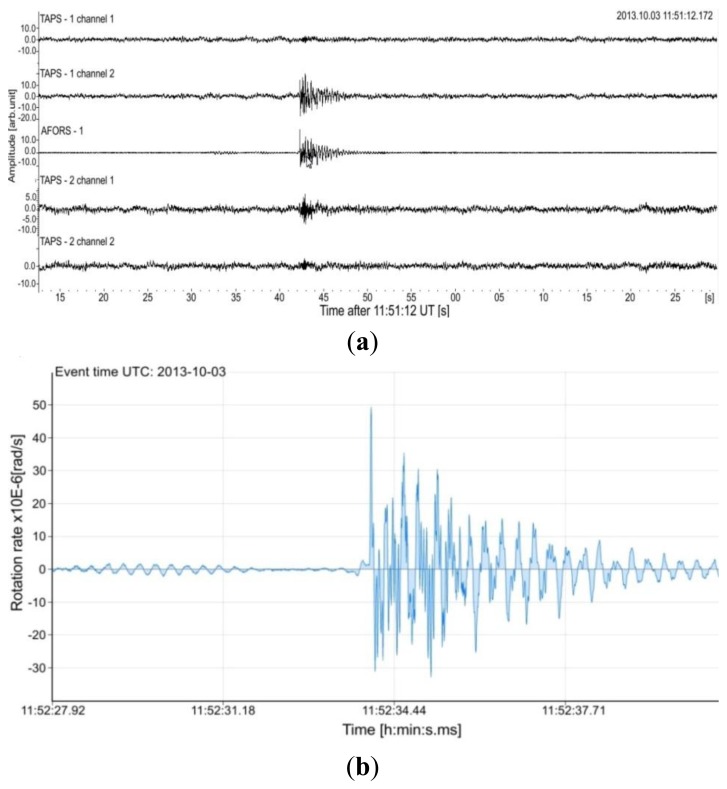
Plots of seismic events recorded in Książ, Poland on 3 October 2013 starting at 11:51 UT. (**a**) Waveforms from KST; (**b**) Seismic rotational event obtained from the FORS-Telemetric Server.

Additionally, in Table 1 the main information connected with the plots illustrated in Figures 4 and 5b, collected by FORS-Telemetric Server are presented. The first of them—ADEV—presents the deviation of the average measured rotation rate Ω for recording seismic events which is used as a noise reference level (see relation (4b)). The second one—Omega Offset—presents the measured offset of Ω. This value is used for the correction of the beginning plot to null level. The third parameter GS Level is connected with the chosen threshold level (*k_L_* multiplied by ADEV). Finally, the last parameter presents the detection frequency band which was chosen in discrete form in the range from 0.83∼106.15 Hz.

In practice, two types of sensors for detection rotational seismic events are mounted at Książ-AFORS and TAPS. Such a situation allows one to compare their recordings. Figure 5a presents the example of data obtained jointly for AFORS-1 and two TAPSs by the KST recording system, which protects a frequency band equal to 50 Hz. The presented KST record comprises five channels (channels 1 and 2 for TAPS-1, channel 3 for AFORS-1, channels 4 and 5 for TAPS-2). For TAPS all of them are seismograms of linear motion connected with seismic events. The rotational component can be found by additional mathematical procedures broadly widely in [24,25]. Whereas the AFORS-1 record presented at KST represents directly the rotational component. Moreover, the KST system only gives qualitative information about rotational seismic events without scaling their amplitude, whereas the record from the FORS-Telemetric Server presented in Figure 5b gives quantitative information. This example confirms the usefulness of the constructed AFORS systems.

## 4. Conclusions

The presented AFORS-1 system seems to be a promising device because it provides an opportunity for detecting absolute seismic rotation rates. In this way this system based on the Sagnac effect seems to be a unique device which can detect seismic rotational phenomena in a direct way and it seems that the data obtained this way are clear enough for an identification of seismic rotational motions. Moreover, the FORS-Telemetric Server allows one to change all the necessary AFORS operation parameters remotely via the Internet. To our knowledge, other devices based on different physical principles need an additional data processing step which can be a source of measurement errors. The results obtained during the AFORS-1 calibration suggest its linear accuracy goes down to 5.1 × 10^−9^ to 5.5 × 10^−8^ rad/s. The first value is obtained for a detection frequency band of 0.83 Hz, whereas the second is for 106.15 Hz.

For the above reasons, we decided to conduct a field test by implementation of the AFORS-1 unit in the seismological observatory located at Książ, Poland. The collected data regarding rotational seismic events are currently under processing and will be published in articles devoted to seismology. The main goal of this paper is only the presentation of the system possibilities and the identification of its practical usefulness.

In the authors'opinion the AFORS is one of the limited examples, beside FOG, of fibre-optic interferometric sensors which have been used for field tests for a long time. It should be underlined that during more than three years of its exploitation, whenever we needed to go and adjust it, all work could be done remotely via the Internet and this aspect is also very positive.

## Figures and Tables

**Table 1.** The main parameters of detected events presented in Figures 4 and 5b collected by the FORS-Telemetric Server.

**Figure No.**	**Date of Recorded Event [year-month-day]**	**ADEV [rad/s]**	**Omega Offset [rad/s]**	**GS Level [rad/s]**	**Detection Frequency Band [Hz]**
4a	2011-03-11	4.11 × 10^−8^	5.51 × 10^−5^	6.57 × 10^−7^	21.23
4b	2013-10-02	9.76 × 10^−8^	6.42 × 10^−5^	1.56 × 10^−6^	21.23
4c	2011-11-23	6.45 × 10^−7^	5.98 × 10^−5^	9.67 × 10^−6^	106.15
5b	2013-10-03	1.34 × 10^−7^	6.42 × 10^−5^	2.15 × 10^−6^	21.23

## References

[1] Lee W.H.K., Celebi M., Todorovska M.I., Igel H. (2009). Introduction to the special issue on rotational seismology and engineering applications. Bull. Seismol. Soc. Am..

[2] Kozak J.T. (2009). Tutorial on earthquake rotational effects: Historical examples. Bull. Seismol. Soc. Am..

[3] Droste Z., Teisseyre R. (1997). Rotational and displacemental components of ground motion as deduced from data of the azimuth system of seismographs. Publ. Inst. Geophys. Pol. Acad. Sci..

[4] Eringen A.C. (1999). Microcontinuum Field Theories: Volume 1, Foundations and Solids.

[5] Teisseyre R., Boratynski W. (2002). Continuum with self-rotation nuclei: Evolution of defect fields and equations of motion. Acta Geophys.

[6] Teisseyre R. (2005). Asymmetric continuum mechanics: Deviations from elasticity and symmetry. Acta Geophys.

[7] Teisseyre R., Białecki M., Górski M. (2005). Degenerated mechanics in a homogeneous continuum: potentials for spin and twist. Acta Geophys.

[8] Teisseyre R., Górski M. (2009). Transport in fracture processes: Fragmentation and slip. Acta Geophys.

[9] Teisseyre R., Nagahama H. (1999). Micro-inertia continuum: Rotations and semi-waves. Acta Geophys. Pol..

[10] Jaroszewicz L.R., Krajewski Z., Solarz L., Marc P., Kostrzyński T. A New Area of the Fiber-Optic Sagnac Interferometer Application.

[11] Sagnac G. (1913). The light ether demonstrated by the effect of the relative wind in ether into a uniform rotation interferometer. Acad. Sci..

[12] Schreiber U., Schneider M., Rowe C.H., Stedmanand G.E., Schluter W. (2001). Aspects of ring lasers as local earth rotation sensors. Surv. Geophys.

[13] Jaroszewicz L.R., Krajewski Z., Solarz L., Teisseyre R., Takeo M., Majewski E. (2006). Absolute Rotation Measurement Based on the Sagnac Effect. Earthquake Source Asymmetry, Structural Media and Rotation Effects.

[14] Takeo M., Ueda H., Matzuzawa T. (2002). Development of a High-Gain Rotational-Motion Seismograph.

[15] Nigbor R.L., Evans R.J., Hutt C. (2009). Laboratory and field testing of commercial rotational seismometers. Bull. Seismol. Soc. Am..

[16] Jaroszewicz L.R., Krajewski Z., Kowalski H., Mazur G., Zinówko P., Kowalski J.K. (2011). AFORS autonomous fibre-optic rotational seismograph: Design and application. Acta Geophys.

[17] Berg R.A., Lefevre H.C., Shaw H.J. (1981). All-single-mode fiber-optic gyroscope with long-term stability. Opt. Lett..

[18] Böhm K., Marten P., Standigel L., Weidel E. Fiber-Optic Gyro with Digital Data Processing.

[19] Post E.J. (1967). Sagnac effect. Rev. Mod. Phys..

[20] Krajewski Z. (2005). Fiber-Optic Sagnac Interferometer as System for Rotational Phenomena Investigation Connected with Seismic Events. Ph.D Thesis.

[21] Krajewski Z., Jaroszewicz L.R., Solarz L. Optimization of Fiber-Optic Sagnac Interferometer for Detection of Rotational Seismic Events.

[22] Dai X., Zhao X., Cai B., Yang G., Zhou K., Liu C. (2002). Quantitative analysis of the Shupe reduction in a fiber optic Sagnac interferometer. Opt. Eng..

[23] Teisseyre R., Takeo M., Majewski E. (2006). Earthquake Source Asymmetry, Structural Media and Rotation Effects.

[24] Teisseyre R., Suchcicki J., Teisseyre K.P., Wiszniowski J., Palangio P. (2003). Seismic rotational waves: Basic elements of theory and recording. Ann. Geophys.

[25] Solarz L., Krajewski Z., Jaroszewicz L.R. (2004). Analysis of seismic rotations detected by two antiparallel seismometers: Spline function approximation of rotation and displacement velocities. Acta Geophys. Pol..

